# Ring-Opening Polymerization of ε-Caprolactone Initiated by Ganciclovir (GCV) for the Preparation of GCV-Tagged Polymeric Micelles

**DOI:** 10.3390/molecules20022857

**Published:** 2015-02-10

**Authors:** Alicia J. Sawdon, Ching-An Peng

**Affiliations:** Department of Chemical Engineering, Michigan Technological University, Houghton, MI 49931, USA; E-Mail: ajsawdon@mtu.edu

**Keywords:** chitosan, ganciclovir, poly(caprolactone), ring-opening polymerization

## Abstract

Ganciclovir (GCV) is a nucleoside analogue with antiviral activity against herpes viral infections, and the most widely used antiviral to treat cytomegalovirus infections. However, the low bioavailability and short half-life of GCV necessitate the development of a carrier for sustained delivery. In this study, guanosine-based GCV was used as the initiator directly in ring-opening polymerization of ε-caprolactone (ε-CL) to form hydrophobic GCV-poly(caprolactone) (GCV-PCL) which was then grafted with hydrophilic chitosan to form amphiphilic copolymers for the preparation of stable micellar nanoparticles. Successful synthesis of GCV-PCL and GCV-PCL-chitosan were verified by ^1^H-NMR analysis. Self-assembled micellar nanoparticles were characterized by dynamic light scattering and zetasizer with an average size of 117 nm and a positive charge of 24.2 mV. The drug release kinetics of GCV was investigated and cytotoxicity assay demonstrated that GCV-tagged polymeric micelles were non-toxic. Our results showed that GCV could be used directly in the initiation of ring-opening polymerization of ε-CL and non-toxic polymeric micelles for GCV delivery can be formed.

## 1. Introduction

Ganciclovir (GCV), is the most widely used antiviral drug for the treatment of human cytomegalovirus (CMV) infections [1]. GCV however is only slightly water soluble, and as a result, has poor oral and ocular bioavailability. Therefore, it needs to be administered intravenously; however, due to its short biological half-life, frequent dosing is required, which may increase the risk of systemic toxicity and discomfort to the patient [1,2,3,4]. To address the aforementioned problems, several drug delivery systems have been developed for GCV [5,6,7,8,9]. Incorporation of the drug into nanoparticles is advantageous because a carrier can protect the drug from enzymatic degradation, control the drug release rate (leading to enhanced bioavailability), improve the therapeutic effects, and reduce administration frequency.

In recent years, polymeric micelles have been the focus of much interest as alternative vehicles for the solubilization of poorly water-soluble molecules rendering clear advantages over current solubilizing agents in drug delivery [10]. Polymeric micelles are expected to withstand the diluting effect of blood, stay in a micellar form, and even act as a circulating depot drug delivery system after intravenous administration. Moreover, there is no inclusion of potentially harmful surfactants and excipients in the process of encapsulating hydrophobic drug molecules. Due to the fact that micelles can be specifically synthesized to increase a drug’s solubility and bioavailability, they are a model system for enhanced drug delivery [11,12,13,14].

Poly(ε-caprolactone) (PCL) having been widely used as the core-forming hydrophobic segment of nanoparticles was selected as the model polymer for this study. PCL is a semi-crystalline linear resorbable aliphatic polyester. It has been commonly used in drug delivery systems because it is biodegradable and biocompatible [15,16,17]. PCL is commonly synthesized by ring-opening polymerization of ε-caprolactone using an alcohol (R-OH) as an initiator and stannous (II) octoate (Sn(Oct)_2_) as a catalyst [18,19]. In addition to using R-OH as the initiator, methoxy-poly(ethylene oxide) and starch have been employed as macroinitiators to form amphiphilic polymers [20,21]. In this study, prodrug GCV possessing hydroxyl groups, was used as the initiator to obtain GCV-PCL. Then, hydrophilic chitosan was grafted on the hydrophobic GCV-PCL to form the amphiphilic block copolymer which already has the GCV prodrug attached started from the ring-opening polymerization. Chitosan is a natural polysaccharide derived from deacetylation of chitin. Chitosan’s biocompatible and biodegradable features have attracted much attention in biomedical and pharmaceutical research [15,21].

The synthesized GCV-tagged amphiphilic copolymer can be self-assembled in aqueous medium to form polymeric prodrug micelles, which can be used further to treat CMV infections. To this end, the chemical structure and physical properties of GCV-PCL-chitosan were characterized and polymeric prodrug micelle formation was investigated. Drug release of GCV from GCV-PCL-chitosan, as well as the biocompatibility of GCV-tagged polymeric micelles were examined.

## 2. Results and Discussion

### 2.1. Synthesis and Characterization of Amphiphilic Prodrug Polymers

**Scheme 1:**
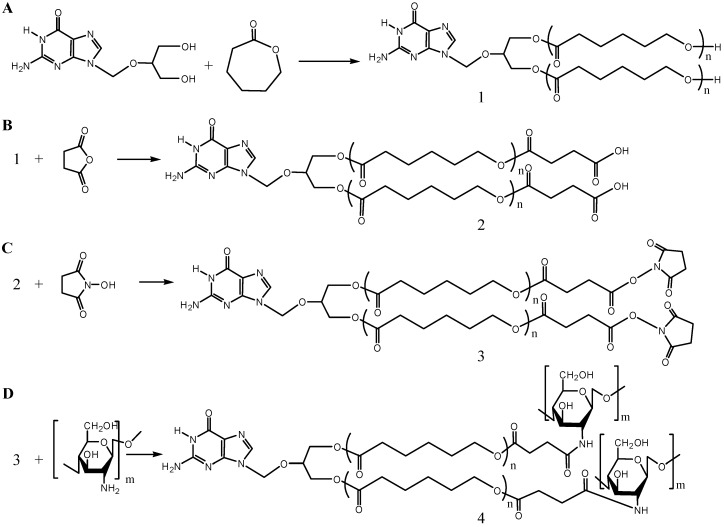
Synthetic steps of (**A**) GCV-PCL, (**B**) GCV-PCL-COOH, (**C**) GCV-PCL-NHS, and (**D**) GCV-PCL-chitosan.

**Figure 1:**
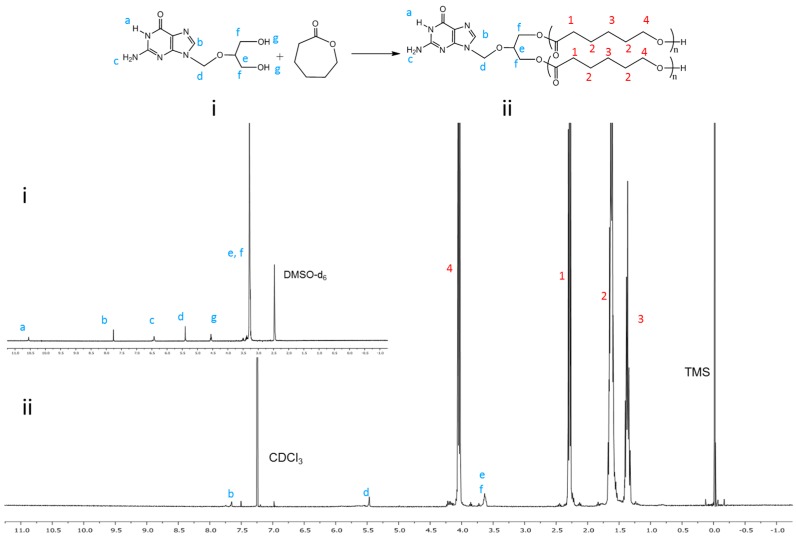
^1^H-NMR spectra of (**i**) GCV and (**ii**) GCV-PCL.

GCV-PCL was synthesized through ring-opening polymerization of ε-CL exclusively by GCV (Scheme 1A) using Sn(Oct)_2_ as a catalyst. While the mechanism of polymerization with Sn(Oct)_2_ is still unclear, Sn(Oct)_2_ is not thought to be the actual initiator since polymer molecular weight does not depend on the monomer-to-Sn(Oct)_2_ molar ratio [18]. The most widely accepted mechanism for ring-opening polymerization is a coordination insertion mechanism where the hydroxyl functional group coordinates to Sn(Oct)_2_ forming the initiating tin-complex. To show the successful initiation of ε-CL by GCV, ^1^H-NMR analysis was conducted. The ^1^H-NMR spectra of prodrug GCV and GCV-PCL are shown in Figure 1i,ii, respectively. Chemical shifts at δ = 1.37 (j-CH_2_), 1.62 (i-CH_2_), 2.27 (h-CH_2_) and 4.04 (k-CH_2_) ppm correspond with the backbone chain of PCL polymer. Peaks at δ = 3.63 (f-CH_2_), 5.47 (d-CH_2_) and 7.76 (b-CH) are assigned to protons in GCV. Evidence of GCV grafting to PCL is clearly seen by the characteristic resonances observed in the synthesized polymer, confirming the synthesis of GCV-PCL.

**Figure 2:**
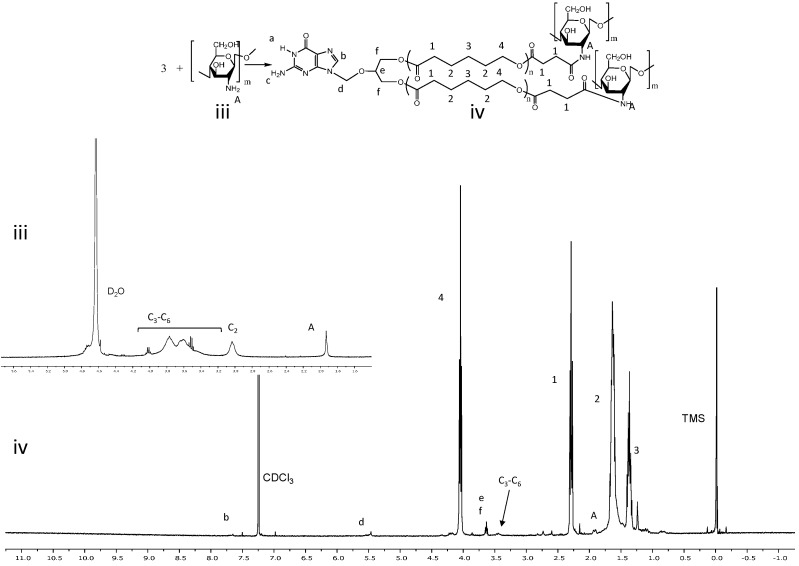
^1^H-NMR spectra of (**iii**) chitosan and (**iv**) GCV-PCL-chitosan.

**Figure 3:**
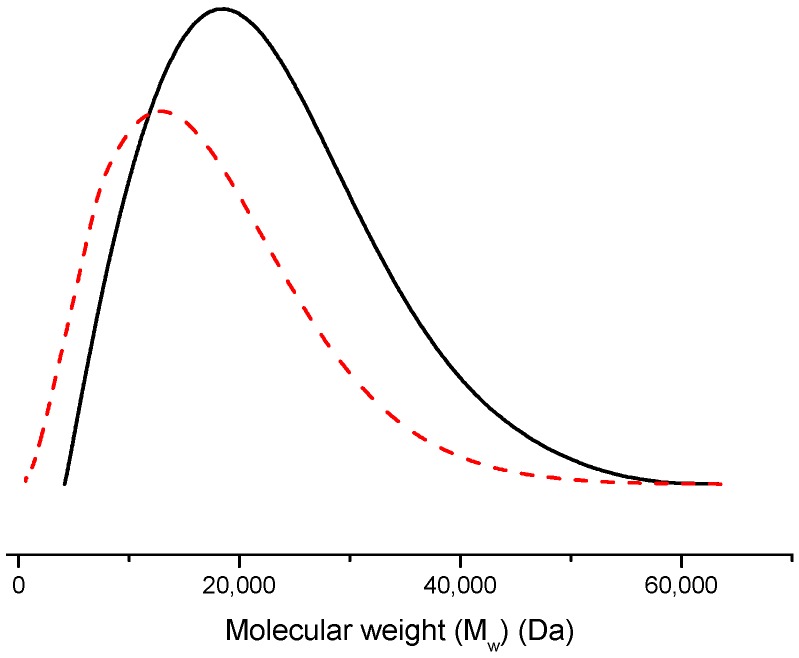
Characterization of molecular weight profiles of GCV-PCL (---) and GCV-PCL-chitosan (^____^) by GPC analysis.

GCV-PCL was further conjugated with chitosan as shown in Scheme 1B–D. Successful conjugation of chitosan was confirmed by analytical means. Figure 2 depicts the ^1^H-NMR analysis of chitosan (iii) and GCV-PCL-chitosan (iv). As shown in Scheme 1D, conjugation of chitosan to GCV-PCL was made via amide linkage. The peak at 1.79 (l-NH_2_) from a singlet to a multiplet in Figure 2iv confirms conjugation of chitosan to GCV-PCL. Moreover, the peaks from the protons on C_3_–C_6_ of chitosan can be seen from δ = 3.28–3.85, slightly shifted downward from the peaks shown in the original chitosan sample (Figure 2iii). Moreover, the gel permeation chromatography (GPC) data shown in Figure 3 confirmed the formation of amphiphilic copolymer GCV-PCL-chitosan. GCV-PCL hydrophobic polymer had an observed number-average molecular weight (M_n_(obsd)) of 11.5 kDa which increased to 17.2 kDa after the addition of chitosan. This corresponds well with the addition of chitosan which had an average molecular weight of 5 kDa. Furthermore, the polydispersity index (PDI) of both GCV-PCL and GCV-PCL-chitosan polymer was low at 1.13 and 1.18, respectively which indicate that the polymer chains are approaching a uniform chain length. The molecular weights of GCV-PCL and GCV-PCL-chitosan were found via GPC calibrated by polystyrene standards. The data were corrected via Mark-Houwink correction for M_n_(corrected) = 0.56 × M_n_(obsd) and the data are shown in Figure 3 [22,23].

**Table:** 

Sample	M_w_ (Da)	M_n_(obsd) ^a^ (Da)	M_n_(corrected) ^b^ (Da)	Polydispersity (M_w_/M_n_(obsd))
GCV-PCL	12,996	11,454	6414	1.13
GCV-PCL-chitosan	20,354	17,231	9649	1.18

Notes: ^a^ Measured by GPC in THF relative to polystyrene standards; ^b^ M_n_ calculated after Mark-Houwink correction: M_n_(corrected) = 0.56 × M_n_(obsd).

### 2.2. Formation and Characterization of GCV-Tagged Polymeric Micelles

**Figure 4:**
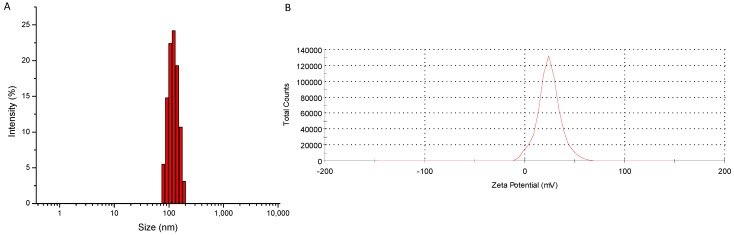
Physical characterization of GCV-PCL-chitosan micelles, (**A**) size analysis reported by DLS and (**B**) charge reported by zetasizer.

Through the solvent evaporation method, polymeric micelles of GCV-PCL-chitosan were formed. Here, the hydrophobic core segment was GCV-PCL and chitosan was the cationic and hydrophilic corona segment. The size of GCV-PCL-chitosan micelles was examined through dynamic light scattering (DLS) analysis. Figure 4A shows the size of GCV-tagged polymeric micelles. The average size as reported by DLS was 117 nm with a zeta potential of 24.2 mV (Figure 4B). The positive charge is attributed to chitosan used as the hydrophilic segment on the micellar carriers. To determine the drug loading percentage of GCV per mg of prepared micelle solution, the absorbance of GCV-PCL-chitosan was examined at t = 0 and t = 72 h and compared to a standard calibration curve of GCV ranging from 0.002 to 1.0 mg·mL^−1^. It was found that GCV comprised 4.8% of the polymeric micelles.

### 2.3. In Vitro Release of GCV from Polymeric Micelles

**Figure 5:**
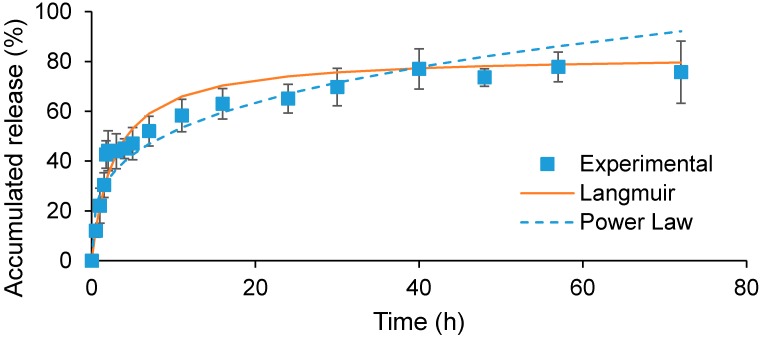
*In vitro* drug release profile of GCV from GCV-PCL-chitosan in PBS at 37 °C (mean ± SD, *n* = 3).

The *in vitro* release behavior of GCV at 37 °C in PBS was examined. As can be seen from Figure 5, the release of GCV took 40 h to reach a maximum release of 77%. Moreover, there was an initial burst release within the first 2 h, followed by sustained release until 40 h. It is surmised that this burst release is due to the release of GCV which may already have weakened ester bonds between GCV and PCL. This is due to the fact that in order to bind chitosan to GCV-PCL-NHS, aqueous solvent is needed to dissolve chitosan, which can begin the breakage of ester bonds via hydrolysis. When conjugating GCV-PCL-NHS with methoxypolyethylene glycol amine (MPEG-NH_2_) in an organic solvent, the burst release of GCV from prepared GCV-PCL-MPEG micelles was not observed as the one shown in Figure 5 (data not shown). The release kinetics of GCV from GCV-PCL-chitosan was modeled using both power-law and Michaelis-Menten-type models. As can be seen from Figure 5, power-law model was not a good fit for the release of GCV from polymeric micelles. Here, the exponent, *n*, was equal to 0.29 in GCV-PCL-chitosan micelles. The exponent, *n*, of a sphere with Fickian diffusion as the drug release mechanism is 0.43 according to theoretical calculation [24]. Because our *n* value is lower than this value, the drug release mechanism of GCV from polymeric micelles is not solely through diffusion. Instead, the release of GCV from polymeric micelles was modulated by hydrolysis and diffusion. The ester bond must be first cleaved via hydrolysis and then GCV can diffuse out of the polymeric micelles. In contrast to the power-law model, the Michaelis-Menten model fits the release of GCV from polymeric micelles well because GCV release is driven by a reaction-diffusion mechanism. According to the Michaelis-Menten model, we found that the dissociation constant (K_d_) was equal to 2.79 which is much higher than the release of ACV from ACV-PCL-chitosan polymeric micelles reported previously [25]. Such extended release of GCV from polymeric micelles probably is due to the relatively robust binding force between GCV-PCL generated by two hydroxyl groups existing on GCV, in comparison with only one hydroxyl moiety on ACV.

### 2.4. Cytotoxicity Test

**Figure 6:**
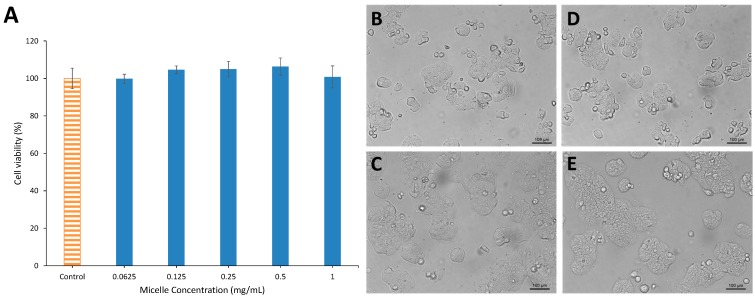
Viability and morphology of HT29 colorectal cancer cells challenged with GCV-PCL-chitosan polymeric micelles at different dosages ranging from 0–1 mg/mL. (**A**) Viability of HT29 cells after 48 h treatment determined by MTT assay; (**B**) untreated cells, 0 h; (**C**) micelle treated cells, 0 h; (**D**) untreated cells, 48 h; (**E**) micelle treated cells, 48 h.

For a micelle carrier to be effective for any sort of drug delivery application, biocompatibility is necessary. To evaluate the toxicity of GCV-PCL-chitosan, HT29 colorectal cancer cells were treated with various concentrations of GCV-tagged polymeric micelles ranging from 0–1 mg/mL. As can be seen in Figure 6A, MTT assay shows that the viability of HT29 cells after 48-h treatment with GCV-PCL-chitosan had little change when compared with the untreated cells (*i.e.*, the control group). Moreover, cell images showed that micelle treatment at 1 mg/mL did not alter the growth morphology of HT29 cells (Figure 6C,E). Figure 6B–E showed that HT29 cells were still able to grow and proliferate after micelle treatment similar to the control group without any micelle challenge. These results demonstrate that GCV-PCL-chitosan micelles were non-toxic and biocompatible.

In summary, we have shown that antiviral GCV can be used as the sole initiator in ring-opening polymerization of ε-CL, forming hydrophobic GCV-PCL. GCV-PCL can be further conjugated with chitosan for the formation of polymeric micelles. Polymeric micelles tagged with GCV have the drug attached to the polymer rather than housed in the core. Our results show that GCV-tagged polymeric micelles are biocompatible and have a small size for therapeutic use.

## 3. Experimental Section

### 3.1. Materials

GCV was purchased from Biotang Inc. (Lexington, MA, USA). *N*,*N'*-dicyclohexylcarbodiimide (DCC), ε-CL, pyrene, and succinic anhydride were purchased from Acros Organics (Geel, Belgium). Sn(Oct)_2_, CDCl_3_ with 1% tetramethylsilane (TMS), deuterated dimethyl sulfoxide (DMSO-*d_6_*), dimethyl sulfoxide (DMSO), tetrahydrofuran (THF), dichloromethane (DCM), methanol, 2-propanol, hexane, toluene, and chitosan oligosaccharide lactate (MW = 5000) were all purchased from Sigma-Aldrich (St. Louis, MO, USA). Ethyl ether was purchased from J.T. Baker (Austin, TX, USA). *N*-Hydroxysuccinimide (NHS) was purchased from Alfa Aesar (Ward Hill, MA, USA). Acetone was purchased from Pharmco-AAPER (Shelbyville, KY, USA). Pyridine and hydrochloric acid (HCl) were purchased from EMD (Philadelphia, PA, USA). Sodium chloride (NaCl) and magnesium sulfate were purchased from Showa (Tokyo, Japan). All reagents were used as received without further purification.

### 3.2. Characterization Methods

Gel permeation chromatography (GPC) analyses were performed on a Waters 1525 binary HPLC pump equipped with a Waters 2414 refractive index detector (Milford, MA, USA). Waters styragel HR 3 (MW = 500–30,000) and HR 4E (MW = 50–100,000) columns were equipped. Molecular weight calibration was performed with polystyrene standards that covered a MW range of 400–4.3 × 10^4^. GPC analyses were performed in THF at a flow rate of 1 mL·min^−1^ with an injected volume of 50 µL. ^1^H-NMR spectra were obtained from a Varian Unity/Inova 400 MHz instrument (Sparta, NJ, USA).

### 3.3. Synthesis of GCV-Tagged Amphiphilic Polymers

GCV (50 mg) was weighed and mixed with ε-CL (2.25 mL) under a sonication bath for 5 min at room temperature. Sn(Oct)_2_ (0.5 wt % of ε-CL) was then added into the mixture. The entire solution was placed into a 3-necked round-bottom flask. The system was purged with nitrogen and immersed in an oil bath at 140 °C for 24 h. The crude product was cooled to room temperature, dissolved in DCM, and precipitated by cold methanol. The product was then vacuum dried by a rotary evaporator at 40 °C.

GCV-PCL (0.5 mmol) and succinic anhydride (1 mmol) were weighed and dissolved in toluene in a 3-necked round-bottom flask. One mmol pyridine was added and the solution was reacted under nitrogen at 70 °C for 48 h. The product was then precipitated by cold hexane, and spun down. The pellet was re-dissolved in DCM and washed twice each with 10% (v/v) HCl and saturated NaCl solution. The organic phase was isolated and dried with magnesium sulfate then filtered. The carboxylated GCV-PCL was recovered by precipitation in cold hexane and then vacuum dried by rotary evaporation at 40 °C.

GCV-PCL-COOH (0.54 mmol) and NHS (2.7 mmol) were weighed and mixed in 15 mL DCM, and then DCC (2.7 mmol) was added. The reaction was run under a nitrogen environment at room temperature for 24 h. The precipitated byproduct 1,3-dicyclohexylurea was removed by vacuum filtration. The filtrate was added into 35 mL diethyl ether and cooled to 4 °C for 4 h to precipitate GCV-PCL-NHS. The precipitate was collected by centrifugation at 3500 rpm for 5 min, washed with 2-propanol and solvent removed by rotary evaporation at 40 °C.

GCV-PCL-NHS (10 mg) was dissolved in 5 mL acetone and slowly added to chitosan solution (20 mg chitosan oligosaccharide lactate dissolved in 25 mL deionized water). The mixture, purged with nitrogen, was stirred in a round-bottom flask for 24 h. The reacted solution was vacuum dried to remove acetone and then lyophilized. The amphiphilic polymer was then dissolved in DCM and dialyzed (MWCO = 6–8 kD, Spectra/Por) against pure DCM to remove unreacted chitosan. GCV-PCL-chitosan was recovered by rotary evaporation at 40 °C.

### 3.4. Preparation of Polymeric Prodrug Micelles

10 mg of GCV-PCL-chitosan was dissolved in 2 mL acetone. The solution was then added dropwise to 10 mL deionized water under sonication. Acetone was removed by rotary evaporation and the final solution was collected by filtering through a 0.45 µm filter.

### 3.5. Size and Charge of Polymeric Prodrug Micelles

The average particle size of polymeric prodrug micelles was determined by a dynamic light scattering (DLS) instrument (Zetasizer Nano ZS, Malvern Instruments, Westborough, MA, USA) equipped with a red laser at a wavelength of 633 nm and scattering angle of 90° at 25 °C. The zeta potential of the micelles dispersed in deionized water was determined with a zeta potential analyzer (Zetasizer Nano ZS).

### 3.6. Drug Release Kinetics

Polymeric prodrug micelles at a concentration of 1 mg·mL^−1^ were made in phosphate buffered saline (PBS) (1 M, pH 7.4) at 25 °C. Two mL of solution was placed in a dialysis tube (Float-A-Lyzer, Spectrum Labs, Rancho Dominguez, CA, USA) with a MWCO of 3.5–5 kD. The dialysis bag was then immersed in 50 mL PBS at 37 °C. At specified time intervals, 5 µL of sample was removed and replaced with fresh PBS. The amount of GCV released was analyzed by a plate reader (BioTek, Winooski, VT, USA) at 254 nm. All experiments were carried out in triplicate.

### 3.7. Cytotoxicity Test

24-well plates were seeded with human colorectal HT29 cells (HTB-38; ATCC, Manassas, VA, USA) suspended in 0.5 mL Dulbecco’s modified Eagles’ medium (DMEM, Corning Cellgro, Manassas, VA, USA) supplemented with 10% fetal bovine serum (FBS, Atlanta Biologicals, Flowery Branch, GA, USA) and 1% penicillin-streptomycin (Sigma) and incubated at 37 °C in 5% CO_2_ balanced with humidified air for 24 h. In each well, 500 µL of 2 mg·mL^−1^ GCV-PCL-chitosan polymeric micelles (filtered by a 0.45 µm filter) was added. After incubation for 48 h, cell viability was assessed using MTT assay. 200 µL of sterile MTT solution in PBS (4 mg·mL^−1^) was added into the culture wells and incubated for 4 h. The medium containing unreacted MTT was removed and 300 µL DMSO was added to dissolve the insoluble purple formazan crystals formed in cellular mitochondria. The absorbance at 590 nm was measured with a plate reader (BioTek) and results were recorded as viability percentage calculated against the control group without micellar challenge. All experiments were carried out in triplicate.

## 4. Conclusions

In conclusion, GCV-PCL-chitosan polymeric micelles were synthesized and characterized. Our results show that ring-opening polymerization of ε-CL can be initiated by guanosine-based prodrug GCV with Sn(Oct)_2_ as the catalyst. GCV-PCL can be easily produced compared to conventional methods, eliminating drug-loading steps, enhancing drug-carrying capacity, and decreasing production cost. Moreover, hydrophilic and cationic chitosan was grafted onto hydrophobic GCV-PCL for the formation of amphiphilic polymer micelles. ^1^H-NMR and GPC analysis confirmed the graft of chitosan to GCV-PCL. Size and zetasizer analysis showed that micelles in the range of 100 nm can be produced with a positive charge. Additionally, HT29 cells were challenged with GCV-PCL-chitosan polymeric micelles and showed no toxicity or inhibition of cell proliferation. This study shows that GCV-PCL-chitosan polymeric micelles are a potential carrier for antiviral drug therapy.
